# Postoperative Fibro-Inflammatory Pseudocyst of the Vesicouterine Space Mimicking a Chronic Hematoma: A Case Report

**DOI:** 10.7759/cureus.115929

**Published:** 2026-09-08

**Authors:** Rizwana Anjum, Sara Zafar, Mohammad Tariq Mahmood, Rizviya Rahman Mafizur Rahman, Anna Bartosinska-Dyc

**Affiliations:** 1 Obstetrics and Gynaecology, American Hospital Dubai, Dubai, ARE; 2 Medical Imaging, American Hospital Dubai, Dubai, ARE; 3 Pathology, American Hospital Dubai, Dubai, ARE

**Keywords:** adhesions, chronic hematoma, chronic pelvic pain, histopathology, laparoscopy, pseudocyst, vesicouterine space

## Abstract

Chronic pelvic pain remains a challenging clinical problem due to its diverse underlying causes and often nonspecific presentation. Although common conditions account for most cases, rare postoperative or inflammatory cystic lesions within the vesicouterine space may mimic more common pelvic pathologies and create significant diagnostic uncertainty. These lesions may demonstrate imaging features that overlap with those of chronic hematomas and endometriotic cysts, further complicating the preoperative diagnosis.

A woman in her mid-30s, gravida 3, para 1, with two previous miscarriages, presented with a several-month history of left lower abdominal and pelvic pain. Pelvic ultrasound demonstrated a cystic lesion with internal echoes located anterior to the lower uterine segment. MRI revealed an ovoid lesion adjacent to the anterior lower uterine segment, with imaging characteristics suggestive of a chronic hematoma. Due to persistent symptoms, laparoscopic exploration was performed. Intraoperatively, dense adhesions were identified between the uterus, cervix, and bladder. Following adhesiolysis and bladder dissection, a cystic lesion containing dense, friable, whitish-yellow material was identified within the vesicouterine space, and the visible cyst wall and its contents were excised. Histopathological analysis demonstrated a fibrous wall without epithelial or mesothelial lining, accompanied by chronic fibro-inflammatory changes, consistent with a pseudocyst. The patient had an uneventful recovery, with significant improvement in her symptoms.

This case demonstrates the imaging overlap between chronic hemorrhagic collections and fibro-inflammatory pseudocysts and highlights the diagnostic challenge posed by uncommon lesions of the vesicouterine space. In this symptomatic patient, despite imaging findings suggestive of a chronic hematoma, a definitive diagnosis was established through surgical exploration and histopathological examination. The report contributes to the limited literature describing pseudocysts involving the vesicouterine space and emphasizes the need to consider postoperative fibro-inflammatory pseudocyst formation in the differential diagnosis of a non-enhancing vesicouterine lesion.

## Introduction

Chronic pelvic pain is a common gynecological complaint with a broad differential diagnosis, most often associated with conditions such as endometriosis, pelvic inflammatory disease, postoperative adhesions, and chronic inflammatory pelvic disorders [1,2]. Imaging modalities, particularly ultrasound and MRI, play a central role in the evaluation of these patients; however, overlapping radiological features of different pelvic pathologies may make preoperative diagnosis challenging [1,2].

Pseudocysts are non-neoplastic, fluid-filled lesions that lack a true epithelial lining and are most commonly recognized in the pancreas. In the pelvis, they are typically encountered as peritoneal inclusion cysts, also referred to as peritoneal pseudocysts, which develop secondary to peritoneal inflammation, adhesions, endometriosis, or prior pelvic surgery [3-5]. These lesions are predominantly observed in women of reproductive age and are strongly associated with previous surgical or inflammatory pelvic conditions.

Radiologically, peritoneal pseudocysts may appear as complex cystic lesions with variable internal characteristics, frequently mimicking adnexal masses, endometriomas, hemorrhagic cysts, or chronic hematomas [5-9]. This overlap in imaging features may cause diagnostic uncertainty and make it difficult to distinguish benign reactive lesions from more common gynecological pathologies. Localized fibro-inflammatory pseudocysts of the vesicouterine space appear to be rarely described in the literature. We report a case of a vesicouterine space pseudocyst presenting with chronic pelvic pain and radiological findings suggestive of a chronic hematoma, highlighting the diagnostic limitations of imaging and emphasizing the importance of surgical and histopathological confirmation.

## Case presentation

A woman in her mid-30s, gravida 3, para 1, with two previous miscarriages, presented to the gynecology outpatient clinic with a several-month history of left lower abdominal and pelvic pain. The pain was dull, mild to moderate in intensity, continuous, with intermittent exacerbations, and partially relieved by simple analgesics. There were no identifiable aggravating factors. She denied fever, weight loss, or urinary or bowel symptoms. Her menstrual cycles were regular, with occasional episodes of heavy menstrual bleeding.

The patient's medical history was significant for polycystic ovary syndrome and endometriosis. Her obstetric history included one previous cesarean section and two prior dilatation and curettage procedures for miscarriage. She had undergone a recent cervical smear, which was reported as normal. Her surgical history included a laparoscopic appendectomy, cholecystectomy, and previous laparotomy for ovarian cyst removal. Family history was notable for melanoma in her father and polycystic kidney disease. On examination, the patient was hemodynamically stable. Abdominal examination revealed mild tenderness in the left lower quadrant on deep palpation. Speculum and bimanual pelvic examinations were unremarkable.

Pelvic ultrasound demonstrated a normal-sized uterus and a well-defined cystic lesion measuring approximately 2.5 × 2.9 cm, with low-level internal echoes, located anterior to the lower uterine segment (Figure 1). Both ovaries were normal in size and morphology, with no adnexal masses.

**Figure 1:**
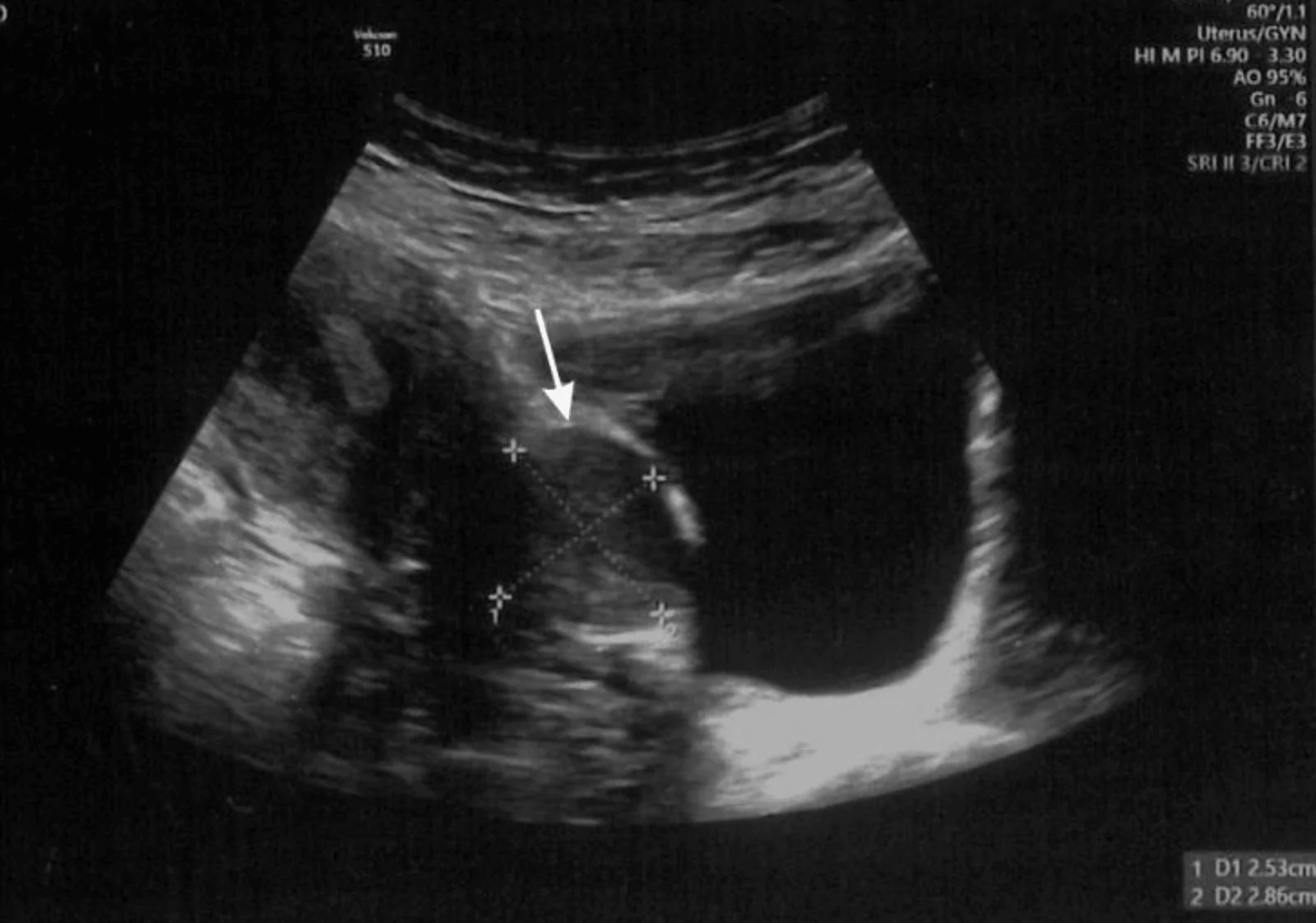
Transabdominal pelvic ultrasound demonstrating a cystic lesion anterior to the lower uterine segment A well-defined 25 × 29 mm cystic lesion with low-level internal echoes is seen anterior to the lower uterine segment (arrow). Based on the ultrasound appearance and previous pelvic surgical history, the differential diagnosis included a hemorrhagic postoperative collection, endometriotic cyst, peritoneal inclusion cyst, and other proteinaceous pelvic cystic lesions

MRI of the pelvis was performed for further characterization. This revealed a 26 × 32 × 36 mm ovoid lesion anterior to the cesarean section scar in the lower uterine segment, adjacent to the anterior cervix. The lesion demonstrated intermediate-to-high signal intensity on T1-weighted images and intermediate-to-low signal intensity on T2-weighted images, with a peripheral T2-hypointense rim (Figure 2). There was no post-contrast enhancement on subtraction T1-weighted fat-saturated images (Figure 3) and no diffusion restriction. These imaging findings were suggestive of a chronic hematoma. The vesicouterine location of the lesion was subsequently confirmed intraoperatively.

**Figure 2:**
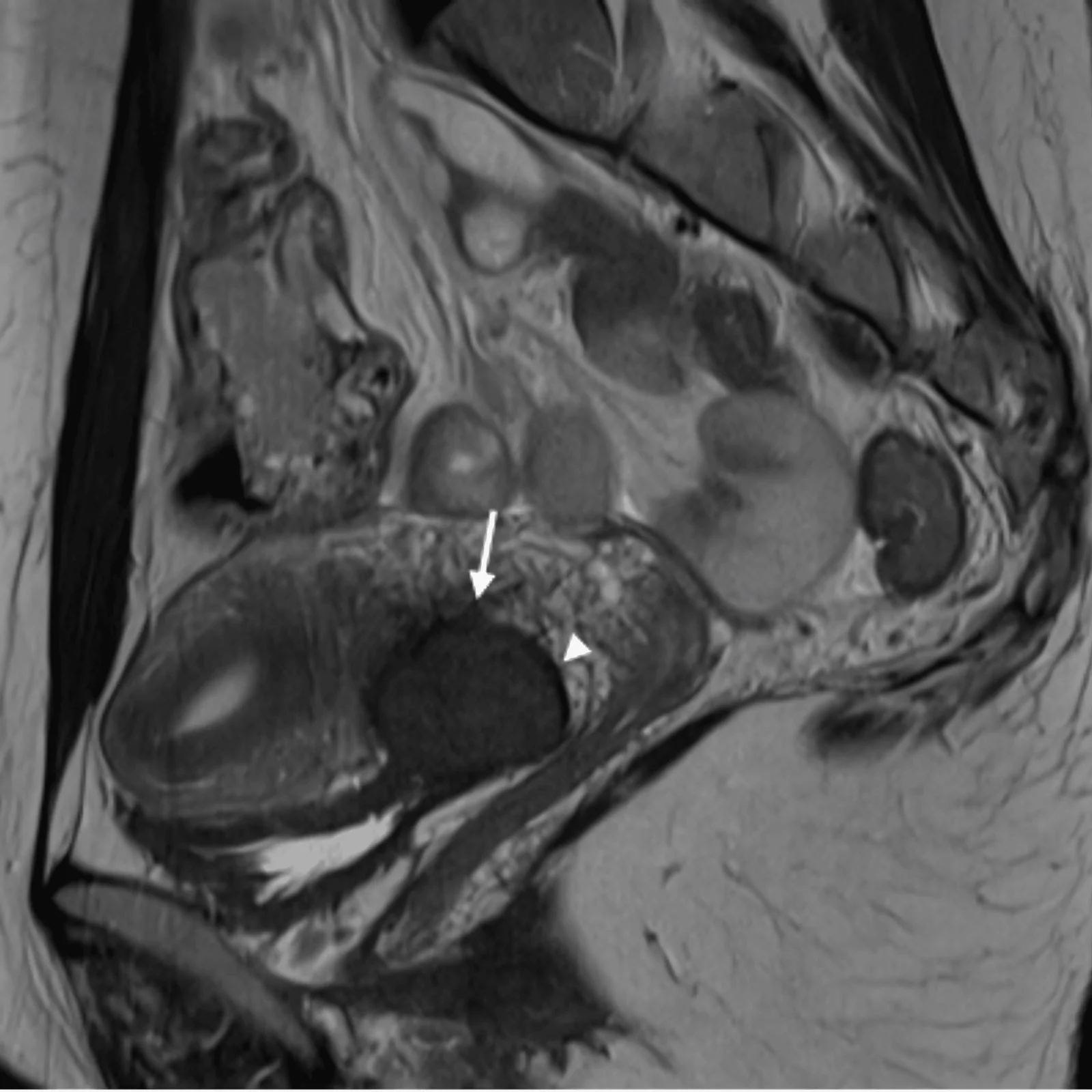
Sagittal T2-weighted pelvic MRI demonstrating a lesion anterior to the lower uterine segment A well-circumscribed lesion is seen anterior to the lower uterine segment and adjacent to the anterior cervix (white arrow). The lesion demonstrates intermediate-to-low T2 signal intensity with a peripheral T2 hypointense rim (arrowhead), suggestive of chronic blood products MRI: magnetic resonance imaging

**Figure 3:**
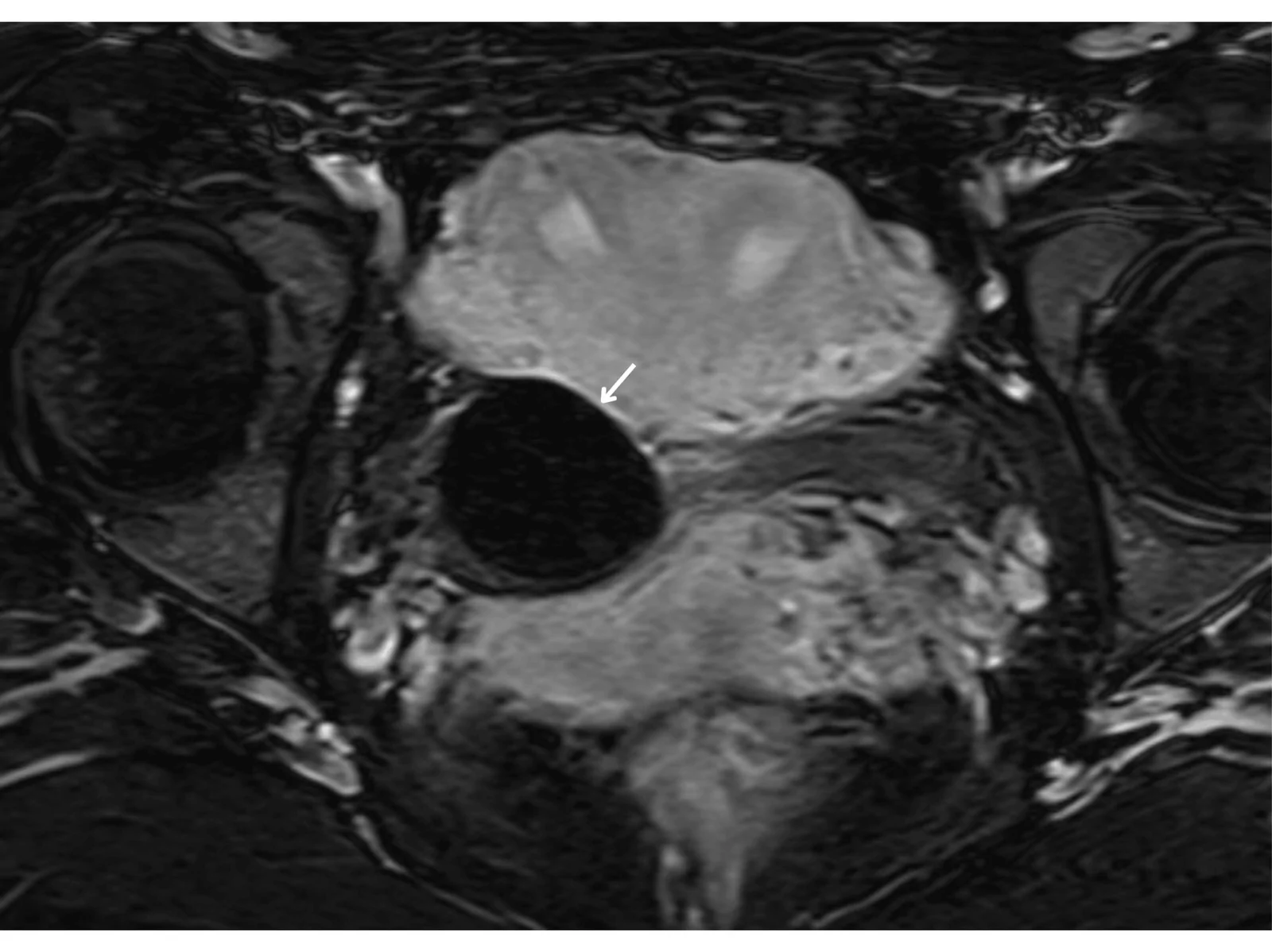
Axial post-contrast pelvic MRI demonstrating a non-enhancing lesion adjacent to the lower uterine segment Axial post-contrast subtraction T1 fat-saturated pelvic MRI demonstrates a well-circumscribed 26 × 32 × 36 mm lesion (white arrow) abutting the lower uterine segment and anterior cervix. No appreciable enhancement is identified following contrast administration, supporting the diagnosis of a chronic hematoma MRI: magnetic resonance imaging

Surgery was undertaken due to persistent symptoms. The patient subsequently underwent laparoscopic exploration and excision of the lesion. Intraoperatively, dense adhesions were noted between the anterior uterine wall, cervix, bladder, and anterior pelvic wall. Following adhesiolysis and bladder dissection, a cystic lesion with a smooth wall and dense internal contents was identified within the vesicouterine space, anterior to the cervix (Figures 4, 5). The cyst was carefully dissected using a combination of blunt and sharp dissection techniques. During dissection, the cyst ruptured, releasing dense, friable, whitish-yellow material. The visible cyst wall and contents were excised and sent for histopathological examination. Bladder integrity was confirmed intraoperatively, and hemostasis was achieved.

**Figure 4:**
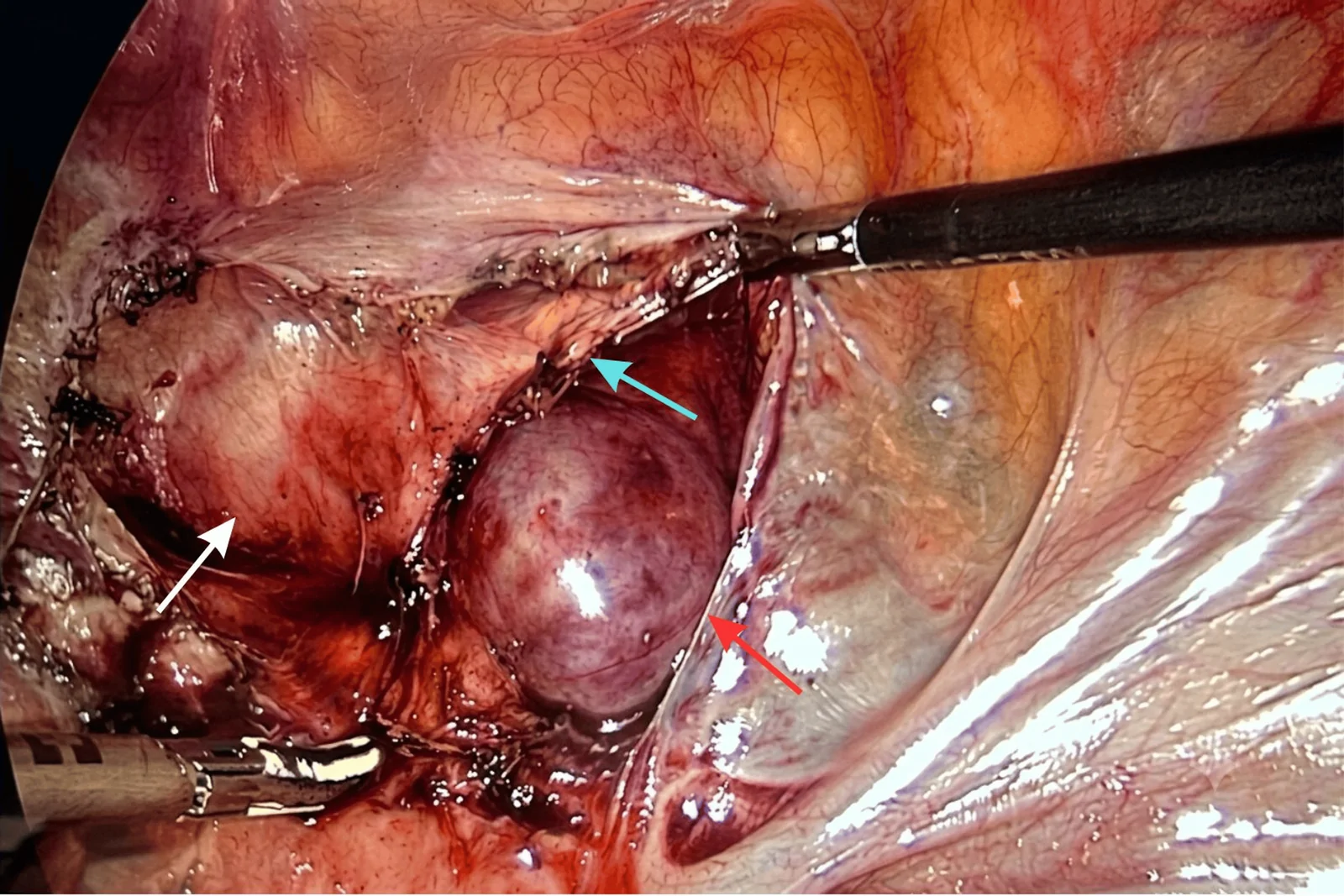
Laparoscopic identification of a cystic lesion within the vesicouterine space Laparoscopic view demonstrates a cystic lesion within the vesicouterine space. The bladder wall (white arrow) is densely adherent to the lower uterine segment and cervix through fibrous vesicouterine adhesions (blue arrow). A well-circumscribed cystic lesion (red arrow) is identified anterior to the cervix during adhesiolysis and bladder dissection

**Figure 5:**
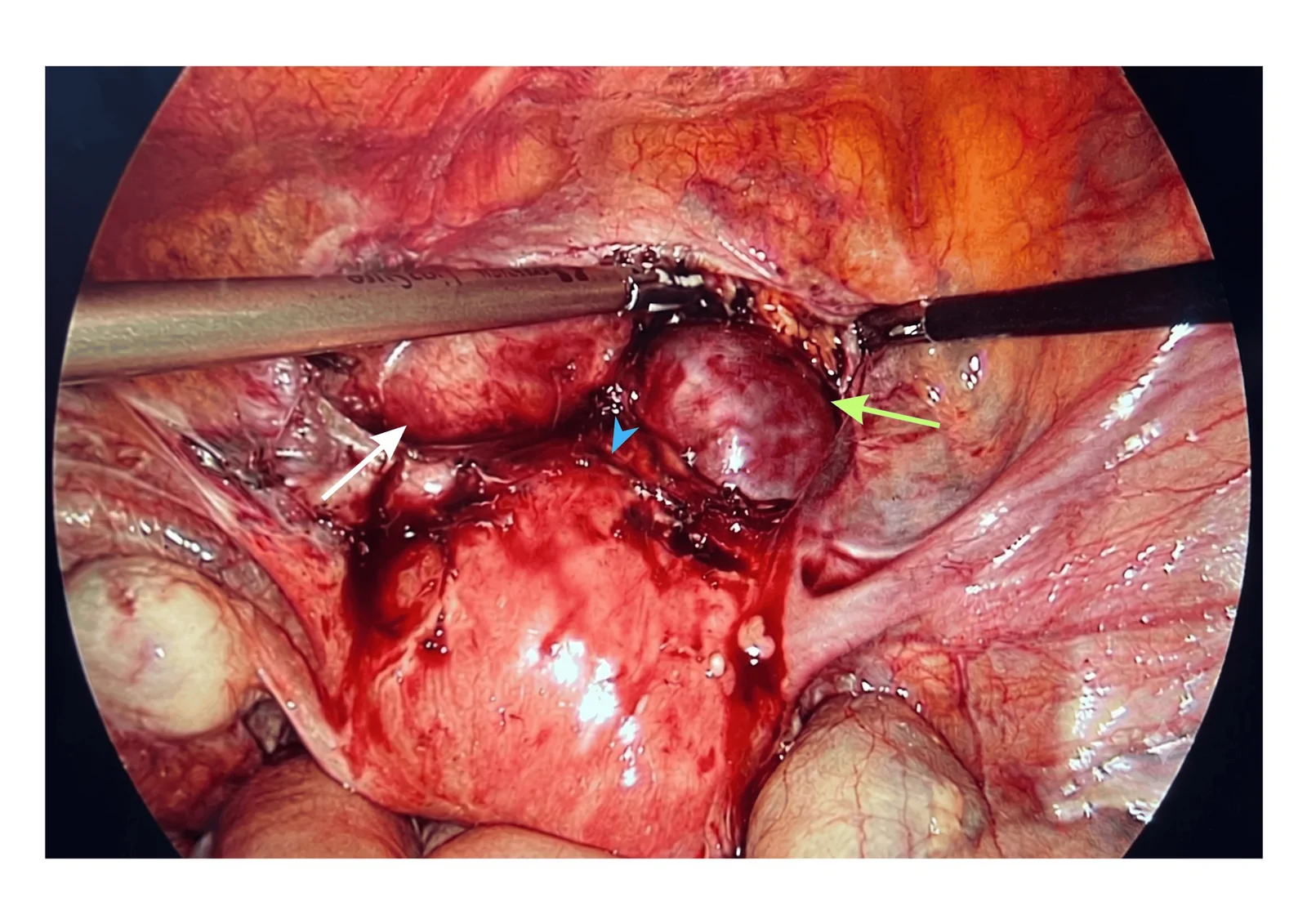
Laparoscopic view demonstrating the cystic lesion in relation to the previous cesarean section scar Laparoscopic view following adhesiolysis demonstrates a cystic lesion (green arrow) within the vesicouterine space anterior to the cervix. The bladder wall is identified superiorly (white arrow). The blue arrowhead indicates the location of the previous cesarean section scar at the lower uterine segment

Histopathological analysis demonstrated a fibrous cyst wall lacking a true epithelial or mesothelial lining, with associated fibro-inflammatory changes, confirming the diagnosis of a pseudocyst (Figures 6, 7).

**Figure 6:**
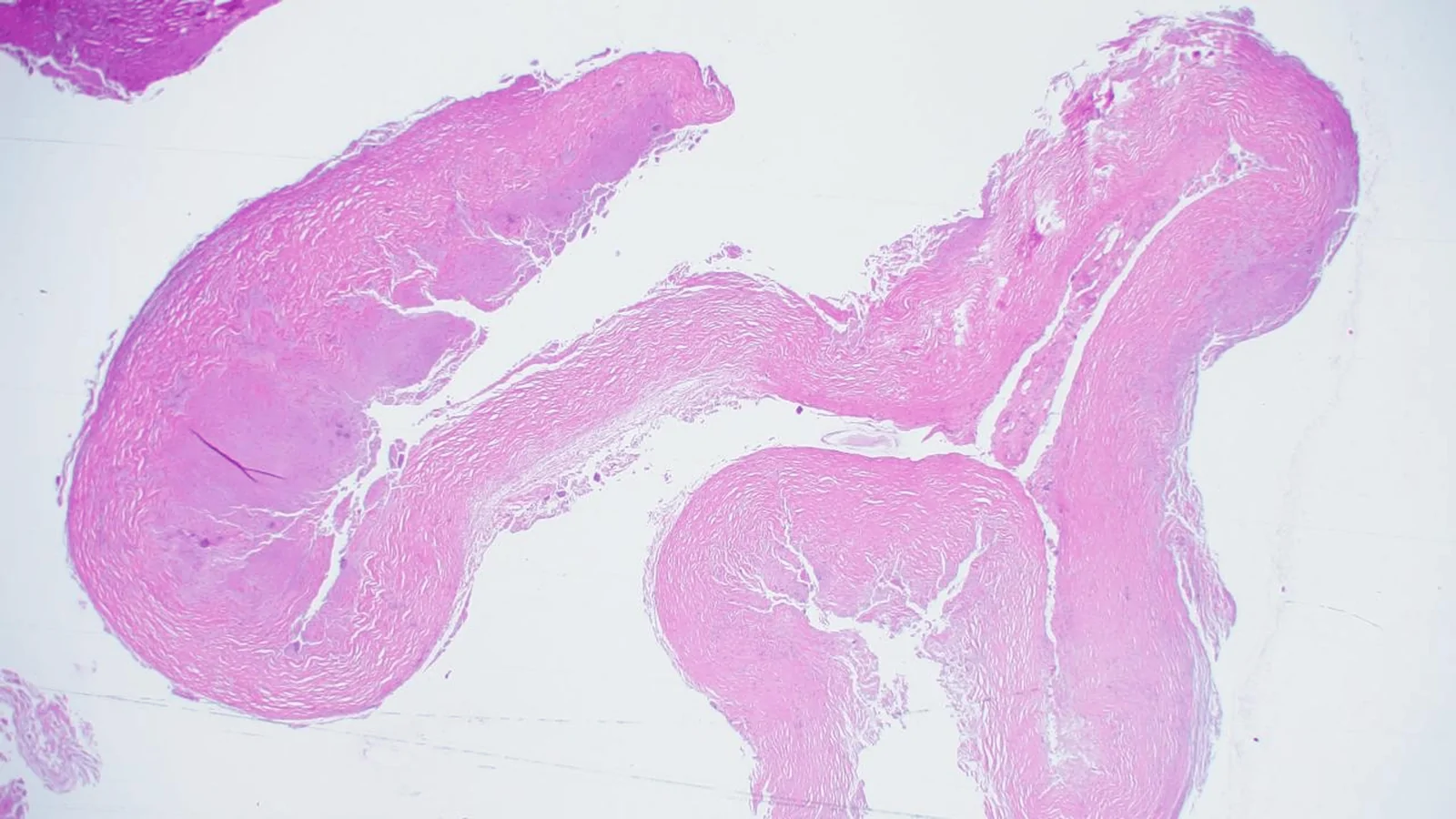
Low-power histopathological appearance of the cyst wall Low-power hematoxylin and eosin (H&E) section demonstrates a benign cyst wall composed predominantly of dense fibrous connective tissue. No epithelial or mesothelial lining is identified, supporting the diagnosis of a fibro-inflammatory pseudocyst

**Figure 7:**
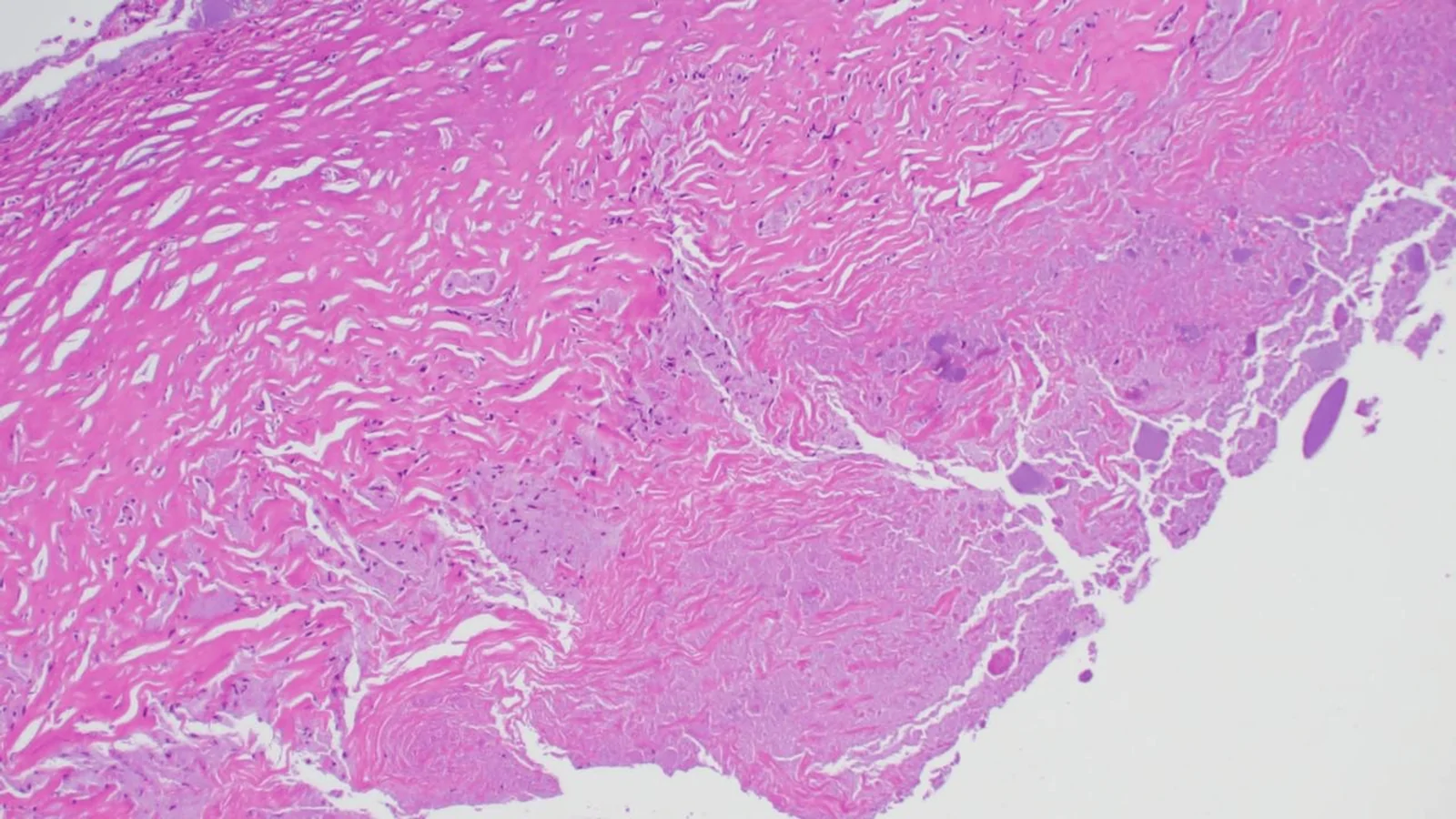
High-power histopathological appearance confirming a pseudocyst Higher-power hematoxylin and eosin (H&E) section shows dense collagenized fibrous tissue with scattered chronic inflammatory cells and focal fibrinous material, without an epithelial or mesothelial lining

The patient had an uneventful postoperative recovery. At the initial three-week follow-up, she reported a marked improvement in her pelvic pain. Clinical follow-up at three and six months demonstrated sustained symptomatic improvement, with no recurrence of pelvic pain.

## Discussion

Chronic pelvic pain presents a significant diagnostic challenge because of its multifactorial etiology, with endometriosis, pelvic adhesions, and postoperative inflammatory changes representing some of the most common underlying causes [1,2]. In the present case, the patient’s history of endometriosis and previous pelvic surgeries raised clinical suspicion for recurrent endometriosis or chronic postoperative sequelae; however, the imaging findings overlapped with those of other pelvic lesions, contributing to preoperative diagnostic uncertainty.

Pseudocysts are characterized histologically by the absence of a true epithelial or mesothelial lining and may develop secondary to prior surgery, chronic inflammation, trauma, hemorrhage, or foreign-body reaction. In the pelvis, these lesions are most commonly described as peritoneal inclusion cysts, resulting from the entrapment of peritoneal fluid within adhesions [3-5]. Peritoneal pseudocysts are strongly associated with prior pelvic surgery, endometriosis, and chronic inflammatory pelvic conditions and are most commonly identified in women of reproductive age [3-5].

Radiologically, pelvic pseudocysts may demonstrate a wide spectrum of imaging appearances, ranging from simple fluid collections to complex cystic lesions with hemorrhagic or proteinaceous content [5,6]. Importantly, these lesions may mimic several gynecological pathologies, including ovarian neoplasms, endometriomas, hemorrhagic cysts, and chronic hematomas [6-9]. In the present case, the patient’s history of endometriosis and previous pelvic surgeries supported the possibility of recurrent endometriosis or chronic postoperative sequelae. However, MRI demonstrated T1 hyperintensity and T2 hypointensity with a peripheral rim and absence of contrast enhancement, findings that initially favored the diagnosis of chronic hematoma. These MRI characteristics may overlap with chronic organized hematoma, endometrioma, and other proteinaceous pelvic lesions, contributing to preoperative diagnostic uncertainty. This highlights the known limitations of imaging in differentiating atypical pelvic cystic lesions, particularly in patients with underlying endometriosis or a history of prior surgery [6,9,10].

Recent literature further highlights the diagnostic challenges associated with postoperative pelvic cystic lesions. Peritoneal inclusion cysts are benign reactive lesions associated with previous abdominal surgery or chronic inflammation and may demonstrate variable appearances on ultrasound and MRI [11]. In addition, cesarean delivery is associated with a spectrum of early and delayed postoperative abnormalities, including pelvic hematomas and scar-related complications, which may pose diagnostic challenges on imaging [12].

Although advances in imaging techniques have improved the evaluation of chronic pelvic pain, definitive diagnosis of atypical pelvic lesions frequently requires surgical exploration and histopathological assessment [3,6]. In this case, laparoscopic excision not only established the diagnosis but also resulted in significant symptomatic improvement. Histopathological examination demonstrated the absence of an epithelial lining, confirming the diagnosis of a pseudocyst. The favorable clinical outcome and sustained resolution of symptoms at six-month follow-up further support surgical excision as an effective management approach for this uncommon lesion.

The distinguishing feature of this case is the unusual localization of the lesion within the vesicouterine space. Most reported pelvic pseudocysts are described as peritoneal inclusion cysts involving the peritoneal cavity or adnexal regions and are commonly associated with prior pelvic surgery, endometriosis, or inflammatory pelvic disease [3-5]. Furthermore, unlike the multiloculated morphology commonly described in peritoneal inclusion cysts, the lesion in this case presented as a solitary, well-circumscribed cystic mass, further contributing to diagnostic uncertainty. Localized fibro-inflammatory pseudocysts of the vesicouterine space appear to be rarely described in the literature.

This report highlights the importance of maintaining a broad differential diagnosis in patients presenting with chronic pelvic pain, particularly in the setting of prior pelvic surgery or inflammatory disease. It also underscores the limitations of imaging in atypical presentations and reinforces the essential role of surgical exploration and histopathological confirmation in establishing a definitive diagnosis.

## Conclusions

Vesicouterine space fibro-inflammatory pseudocysts are rare lesions that may present with chronic pelvic pain and demonstrate imaging features that overlap with those of a chronic organized hematoma. This report highlights the diagnostic limitations of imaging in atypical pelvic lesions and emphasizes the importance of maintaining a broad differential diagnosis in patients with persistent pelvic symptoms. In symptomatic patients in whom imaging findings may mimic other pelvic pathologies, surgical excision and histopathological evaluation remain essential for establishing a definitive diagnosis and guiding appropriate management.
